# Counteracting Fatigue in Multiple Sclerosis with Right Parietal Anodal Transcranial Direct Current Stimulation

**DOI:** 10.3389/fneur.2016.00154

**Published:** 2016-09-21

**Authors:** Katrin Hanken, Mona Bosse, Kim Möhrke, Paul Eling, Andreas Kastrup, Andrea Antal, Helmut Hildebrandt

**Affiliations:** ^1^Department of Psychology, Carl von Ossietzky University Oldenburg, Oldenburg, Germany; ^2^Department of Neurology, Klinikum Bremen-Ost, Bremen, Germany; ^3^Department of Chemistry, Carl von Ossietzky University Oldenburg, Oldenburg, Germany; ^4^Donders Institute for Brain, Cognition and Behaviour, Radboud University Nijmegen, Nijmegen, Netherlands; ^5^University Medical Center, Georg-August University Göttingen, Göttingen, Germany

**Keywords:** multiple sclerosis, vigilance decrement, subjective fatigue, anodal tDCS, right parietal cortex, right frontal cortex

## Abstract

**Background:**

Fatigue in multiple sclerosis (MS) patients appears to correlate with vigilance decrement as reflected in an increase in reaction time (RT) and errors with prolonged time-on-task.

**Objectives:**

The aim of this study was to investigate whether anodal transcranial direct current stimulation (tDCS) over the right parietal or frontal cortex counteracts fatigue-associated vigilance decrement and subjective fatigue.

**Methods:**

In study I, a randomized double-blind placebo-controlled study, anodal tDCS (1.5 mA) was delivered to the right parietal cortex or the right frontal cortex of 52 healthy participants during the first 20 min of a 40-min lasting visual vigilance task. Study II, also a randomized double-blind placebo-controlled study, investigated the effect of anodal tDCS (1.5 mA) over the right parietal cortex in 46 MS patients experiencing cognitive fatigue. tDCS was delivered for 20 min before patients performed a 20-min lasting visual vigilance task.

**Results:**

Study I showed that right parietal stimulation, but not right frontal stimulation, counteracts the increase in RT associated with vigilance decrement. Hence, only right parietal stimulation was applied to the MS patients in study II. Stimulation had a significant effect on vigilance decrement in mildly to moderately cognitively fatigued MS patients. Vigilance testing significantly increased the feeling of fatigue independent of stimulation.

**Conclusion:**

Anodal tDCS over the right parietal cortex can counteract the increase in RTs during vigilance performance, but not the increase in subjective fatigue. This finding is compatible with our model of fatigue in MS, suggesting a dissociation between the feeling and the behavioral characteristics of fatigue.

## Introduction

Several studies on the relation between fatigue and cognitive performance in multiple sclerosis (MS) patients found evidence for a relation between fatigue and vigilance (1–5). Vigilance tasks require an individual to sustain attention during a repetitive monotonous task over a prolonged period of time (6). Usually, detection performance in vigilance tasks declines during time-on-task, which is known as vigilance decrement (7). This vigilance decrement strongly correlates with self-reported fatigue and appears approximately after 20–30 min, reflecting in longer reaction times (RTs) and an increase in errors (8–13). Moreover, vigilance tasks are also highly sensitive to fatigue and sleep deprivation (14, 15).

Previous studies demonstrated that MS patients with fatigue present a larger vigilance decrement than healthy controls (2, 16). Moreover, several studies found correlations between fatigue and vigilance decrement in MS patients (3, 5, 17). Therefore, vigilance tasks appear to be well suited to measure fatigue in MS.

Neuroanatomically, vigilance relies on a right-hemispheric frontal, parietal, thalamic, and brainstem network (18–20). Several functional imaging studies demonstrated an association between vigilant attention and brain activity in right-hemispheric cortical areas, such as the dorsomedial, mid- and ventrolateral prefrontal cortex, the anterior insula, the intraparietal sulcus, and the temporo–parietal junction, as well as in subcortical areas, such as the cerebellar vermis, the thalamus, the putamen, and the midbrain (21). Cognitive neuroscience research has consistently documented an association between decreased activity within this vigilance network and a decrease in vigilance performance with time-on-task (11, 22–24).

Considering MS-related fatigue, its pathophysiology has not been well elucidated. It seems that several factors contribute to fatigue in MS. Fatigue may be a direct consequence of the disease process (primary fatigue) or it may be due to MS-related comorbidities (secondary fatigue). Proposed mechanisms for primary fatigue include immune system dysfunction, neuroendocrine dysregulation, demyelination and axonal loss, gray matter atrophy as well as functional abnormalities within various brain networks (25, 26). Concerning structural and functional changes in the central nervous system, especially changes in right frontal and parietal brain areas, which constitute the vigilance network, have been associated with fatigue in MS patients (27–37).

Transcranial direct current stimulation (tDCS) is a non-invasive technique for modulating neuronal activity (38). Anodal tDCS has been found to enhance neural activity, accompanied by an increase in cerebral blood flow in regions under the anode (39, 40). Consequently, anodal tDCS over brain regions that show decreased activity associated with a vigilance decrement might counteract the performance decrement and the increase in subjective fatigue.

Currently, only few studies have investigated the effect of tDCS on MS-related fatigue (41–43). While some of these studies point to a beneficial effect of stimulation over motor or somatosensory brain regions on subjective fatigue (41, 43), other studies, stimulating the prefrontal cortex, did not find significant effects of tDCS (42). However, these studies investigated the effect of tDCS only on subjective fatigue or fatigue complaints, not on objective fatigue measures, such as the vigilance decrement.

The main objective of this study was to investigate whether anodal tDCS over the right frontal or the right parietal cortex might be able to counteract vigilance decrement in cognitively fatigued MS patients. Several studies point to an association between MS-related fatigue and structural as well as functional changes within right frontal and parietal brain areas that constitute the vigilance network (27–37). Therefore, we assume that anodal, excitability-enhancing tDCS over these brain areas should be able to counteract fatigue-related vigilance decrement in MS patients. We first performed a randomized double-blind placebo-controlled study investigating healthy individuals to check whether right frontal or right parietal stimulation provides the best outcome with respect to vigilance performance (study I). The anodal stimulation with the best outcome was then tested in study II, in which the effect of stimulation on both objective and subjective fatigue was analyzed in cognitively fatigued MS patients.

## Study I

### Methods

#### Study Population

Fifty-two healthy individuals aged between 19 and 44 years participated in this study. Most were students from the Department of Psychology of the University of Bremen. Participants were recruited *via* an internal mail delivery system and postings.

Individuals that were pregnant or had metallic head implants, a history of epilepsy, or any other neurological disease were excluded. The study was approved by the ethical board of the Medical Chamber in Bremen, and written informed consent was obtained from participants.

#### Study Design

To compare the efficacy of anodal tDCS over the right frontal vs. right parietal cortex, we designed a randomized double-blind placebo-controlled study (Table 1). Participants were randomized to receive sham tDCS (*n* = 17), active anodal tDCS over the right parietal cortex (*n* = 18), or active anodal tDCS over the right frontal cortex (*n* = 17). Participants were allotted to a condition using the order of entrance in the study and a previously generated randomization list. Neither the participants nor the director of the study were aware of the stimulation condition. All participants performed a 40-min vigilance task. During the first 20 min of the task, participants received either active anodal tDCS or placebo tDCS. After 20 min, the intervention stopped automatically, and the vigilance task was continued without stimulation. As primary outcome measure, we focused on the progress of RTs and errors in the vigilance task (omissions + commissions) because these measures were frequently found to increase with time-on-task (2, 8, 9). Based on a power calculation for the MS patients, we compared vigilance performance during the first 5 min with that of the last 5 min (2). Information about general fatigue, depression, daytime sleepiness, and sleep quality was assessed to investigate possible associations with performance level.

**Table 1:** **Study design of study I**.

Study I			
Before vigilance task	Visual vigilance task (40 min)		After vigilance task
	First 20 min	Last 20 min	
Assessment of demographic data	Vigilance task with respective stimulation	Vigilance task without stimulation	VAS2
Completion of questionnaires:	Right parietal stimulation; *n* = 18, anode P4		
BDI ESS FSMC FSS PSQI VAS1	Right frontal stimulation; *n* = 17, anode F4 Placebo stimulation; *n* = 17, anode F4 or P4		

#### Visual Vigilance Task

The computer-based visual vigilance task of the test battery for attentional performance [TAP 1.7; (44)] was used. Participants were sitting alone in a darkened room observing a bar on a screen that moved upwards and downwards with changing amplitudes in height. They were instructed to detect obviously larger upward amplitudes by pressing a key as quickly as possible. The frequency of critical stimuli was tuned very low to provide highly monotonous task conditions. The interval between critical stimuli varied randomly between 15,000 and 90,000 ms, with ~6 critical stimuli occurring during each 5 min period of testing. Participants were monitored *via* Skype to ensure that they continuously conducted the task.

Participants first performed a practice trial (with a slightly higher frequency of critical stimuli) for ~1 min to adapt to the task. Time-on-task effects were determined by comparing median RTs and the number of errors in the first (BEGIN) and last (END) 5 min of the vigilance task.

#### Psychological Assessment

Participants rated their current feelings of general fatigue before (VAS1) and after (VAS2) the vigilance task using a 100-mm visual analog scale [(VAS); 0 = no fatigue at all, 100 = maximal imaginable fatigue].

Fatigue was also assessed using self-reported questionnaires, namely the Fatigue Severity Scale [FSS; (45)] and the Fatigue Scale for Motor and Cognitive Functions [FSMC; (46)]. The FSS consists of nine items assessing severity and frequency of fatigue, with higher scores representing stronger fatigue. The FSMC is composed of 20 items and evaluates two main components of fatigue, namely motor and cognitive fatigue. The cutoff score between normal and mild pathological fatigue is 43 for the total scale and 22 for the cognitive and motor scale.

Information about daytime sleepiness, sleep quality, and depression was assessed *via* specific questionnaires. The Epworth Sleepiness Scale [ESS; (47)] was used to assess daytime sleepiness. Sleep quality was determined using the Pittsburgh Sleep Quality Index [PSQI; (48)]. Depressive mood was investigated using the Beck Depression Inventory Scale [BDI; (49)].

#### Stimulation

**Figure 1:**
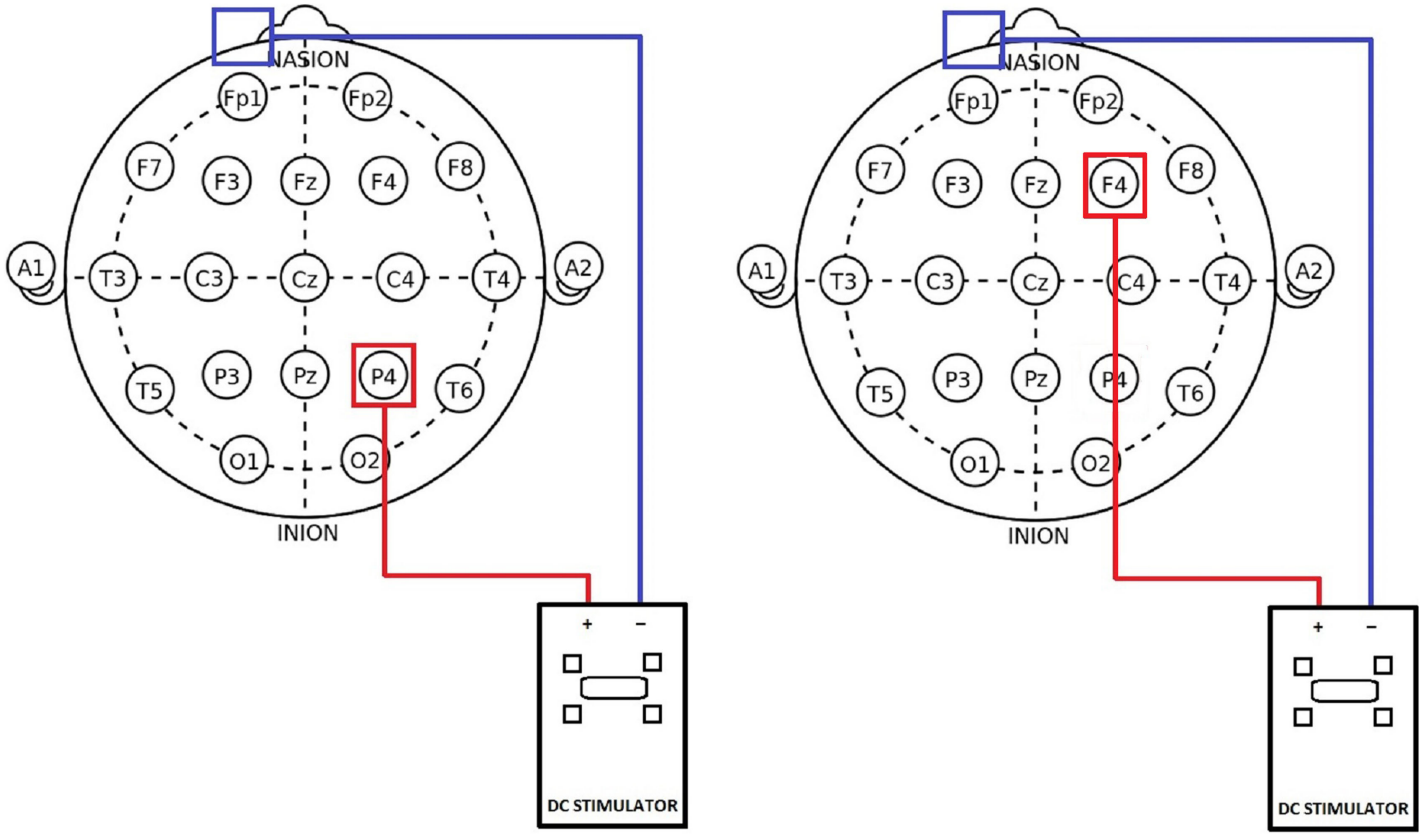
**Position of electrodes in the 10–20 EEG system**. Left: parietal stimulation group with anode (red) located over P4 and the cathode (blue) placed over the contralateral forehead. Right: frontal stimulation group with the anode (red) located over F4 and the cathode (blue) placed over the contralateral forehead.

Anodal tDCS with an intensity of 1.5 mA and with a duration of 20 min was applied using a battery-driven constant current stimulator (DC-Stimulator Plus, NeuroConn, Ilmenau, Germany). The current was ramped up for 30 s until reaching 1.5 mA intensity and ramped down for 30 s at the end of the stimulation. For the placebo stimulation, the current was ramped up for 8 s followed by 30 s of stimulation and a ramping down period of 5 s. During the rest of the placebo intervention period, every 550 ms, a current of 110 μA was released to provide effective blinding. The stimulation electrodes were coated in sponges that were soaked in isotonic saline solution. The anode (5 cm × 7 cm) was either placed over the right parietal cortex (P4 in 10–20 EEG system) or the right frontal cortex (F4 in 10–20 EEG system). The cathode (5 cm × 7 cm) was always placed on the contralateral forehead (current density in the active tDCS conditions = 0.04 mA/cm^2^). In the placebo group, the anode was either located over the right parietal cortex (P4) or over the right frontal cortex (F4). The cathode was invariably placed over the left forehead (Figure 1).

#### Statistical Analysis

Repeated measures analyses of variance (ANOVA) were used to check for group differences in vigilance performance (RT and errors) and subjective fatigue (VAS) using SPSS21 software. The treatment condition (VERUM F4/VERUM P4/PLACEBO) was defined as between-subject factor GROUP. With respect to vigilance performance, we defined TIME (BEGIN/END) as the within-group factor. Concerning subjective fatigue, respective fatigue level before and after the vigilance test were defined as within-group factors TIME (BEFORE/AFTER). Significant main or interaction effects were further tested using *post hoc* tests.

### Results

#### Demographic and Psychological Data

**Figure 2:**
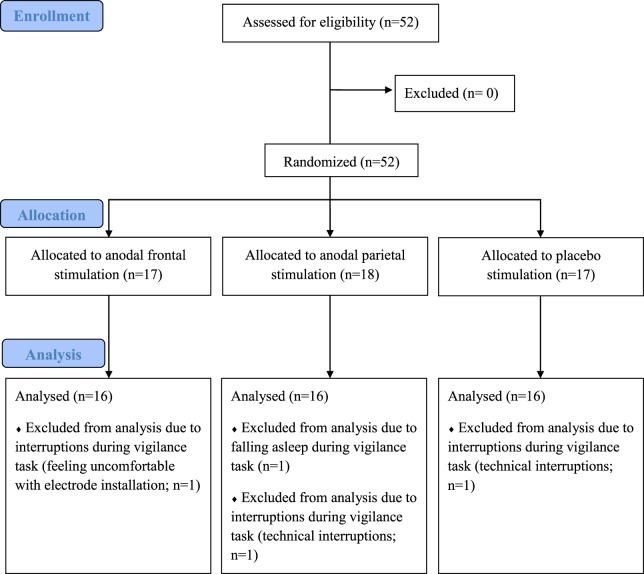
**Flowchart of study I**.

Three participants were excluded from the analyses because investigations were interrupted during the vigilance task. Another participant was excluded because of falling asleep during the vigilance task (Figure 2).

Individuals (*n* = 48) in the three groups did not differ in terms of age, gender, fatigue scores, depression score, or daytime sleepiness. All groups displayed mean FSMC scores suggesting mild pathological fatigue. The placebo group scored significantly lower on the PSQI than the frontal stimulation group (Table 2).

**Table 2:** **Demographic, psychological, and reaction time data of study participants (study I)**.

	Study I		
	Right frontal tDCS	Right parietal tDCS	Placebo tDCS
Number (*n*)	16	16	16
Age (SD)	27.13 (7.71)	26.19 (6.42)	23.75 (2.70)
Gender (male:female)	4:12	4:12	3:13
FSS (SD)	2.98 (0.66)	2.9 (0.94)	3.09 (1.08)
FSMC total score (SD)	47.69 (7.96)	45.81 (8.60)	44.50 (10.56)
FSMC cognitive score (SD)	24.13 (5.62)	22.69 (4.84)	21.75 (5.98)
FSMC motor score (SD)	23.69 (3.42)	23.13 (5.15)	22.75 (5.13)
ESS (SD)	7.75 (2.72)	8.63 (3.90)	8.94 (2.79)
PSQI (SD)*	6.97 (3.26)	5.50 (2.92)	4.59 (2.08)
BDI (SD)	7.75 (4.60)	6.19 (4.63)	6.38 (4.10)
VAS1 (before vigilance task) (SD)	43.73 (24.86)	39.63 (21.25)	40.19 (24.58)
VAS2 (after vigilance task) (SD)	60.44 (28.86)	53.00 (26.21)	57.38 (18.16)
RT (SD) vigilance task first 5 min	438.6 (±115.8)	443.1 (±68.5)	409.8 (±85.5)
RT (SD) vigilance task last 5 min**	489.8 (±116.5)	411.2 (±62.8)	426.4 (±113.5)

*p < 0.05 (significant difference between placebo stimulation and right frontal stimulation group).

**p < 0.05 (significant difference between right frontal and right parietal stimulation group).

*BDI, Beck’s Depression Inventory; FSMC, Fatigue Scale for Motor and Cognition; FSS, Fatigue Severity Scale; ESS, Epworth Sleepiness Scale; PSQI, Pittsburgh Sleep Quality Index; RT, reaction time; VAS Visual Analog Scale*.

#### Performance on the Vigilance Task

**Figure 3:**
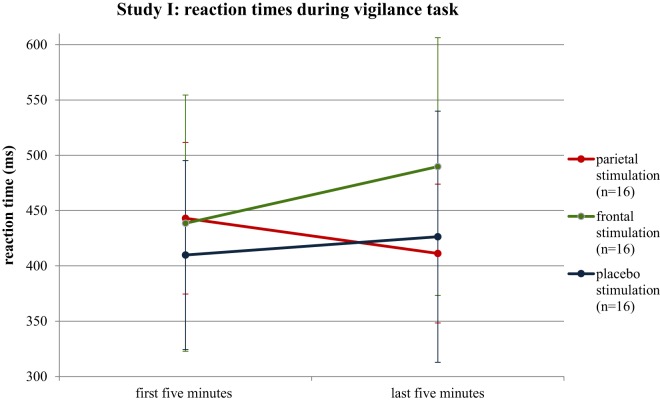
**Median reaction times of the stimulation groups for the first and last 5 min of the vigilance test**. Error bars reflect SDs.

The ANOVA on RTs revealed no main effects, but a significant interaction between GROUP and TIME (*p* = 0.032; *F* = 3.73). The parietal stimulation group showed a decrease in RT with time-on-task, whereas the other two groups presented an increase in RT (Figure 3). *Post hoc* independent *t*-tests revealed no significant group differences in RT during the first 5 min of the vigilance task. In the last 5 min of the vigilance task, a significant difference between the frontal and the parietal stimulation group was observed (*p* = 0.026), with the frontal stimulation group presenting significantly longer RTs. *Post hoc* dependent *t*-tests revealed a significant increase in RT with time-on-task for the frontal stimulation group (*p* = 0.020), whereas the parietal stimulation group showed a moderate decrease in RT (*p* = 0.074; Table 2).

The ANOVA on errors showed no significant effect of GROUP or an interaction with GROUP. However, there was a significant main effect of TIME (*p* = 0.003; *F* = 10.08), with all groups making more errors in the first 5 min than in the last 5 min of the vigilance task. *Post hoc* Wilcoxon tests revealed a significant decrease for the placebo (*p* = 0.019) and the parietal stimulation group (*p* = 0.004).

#### Subjective Fatigue

The analysis of the VAS data before and after the vigilance task revealed a significant main effect of TIME (*p* < 0.001; *F* = 24.8), with all groups showing an increase in the VAS score. *Post hoc* dependent *t*-tests demonstrated that the increase on the VAS was significant for the placebo (*p* = 0.002) and the frontal stimulation group (*p* = 0.005), but not for the parietal stimulation group (*p* = 0.063).

### Discussion

Our results suggest that anodal stimulation of the right parietal cortex exerts a positive effect on the decrement of RT, unlike stimulation over the right frontal cortex that even seems to exert a negative effect on vigilance performance. Subjective fatigue level before and after the vigilance task revealed a significant increase for the placebo and the frontal stimulation group, but not for the parietal group. On the basis of these results, we decided to use only anodal stimulation of the right parietal cortex in study II.

## Study II

### Methods

#### Study Population

Forty-six MS patients with a relapsing–remitting (*n* = 18) or a secondary progressive (*n* = 28) disease course according to the McDonald criteria (50) participated in this study. A required sample size of at least 40 MS patients was determined on the basis of the data of a former study, in which we investigated the effect of a cooling vest on vigilance performance (2). We identified a significance level α of 0.05 and a power of 0.80. The relevant difference was determined as the standardized mean difference of the vigilance performance data.

Patients were recruited from MS support groups or have been patients of the Klinikum Bremen-Ost, Germany. Patients received either disease-modifying drugs (67%) or no therapy (33%). Individuals with a MS relapse or using corticosteroids during the last four weeks, under legal care and/or with a diagnosis of any other neurodegenerative disease were excluded from the study. Further exclusion criteria were the presence of metallic head implants, a history of epilepsy, and pregnancy. The study was approved by the ethical board of the Medical Chamber in Bremen, and written informed consent was obtained from participants.

#### Study Design

We designed a randomized double-blind placebo-controlled study (Table 3). Patients were randomized to receive sham tDCS (*n* = 23) or active anodal tDCS over the right parietal cortex (*n* = 23). Randomization was performed using the order of entrance in the study and a previously generated randomization list. tDCS was applied for 20 min before performing a 20-min visual vigilance task, unlike in study I.

**Table 3:** **Study design of study II**.

Study II				
Before stimulation	Stimulation 20 min	After stimulation	Visual vigilance task 20 min	After vigilance task
Assessment of demographic and clinical data Completion of questionnaires: BDI ESS FSMC FSS PSQI VAS1	Stimulation group; *n* = 18, anode P4 Placebo group; *n* = 18, anode P4	VAS2	Vigilance task without stimulation	VAS3

#### Visual Vigilance Task

The visual vigilance task was identical to that in study I, but lasted 20 min, and was performed directly after a 20-min stimulation period.

#### Psychological and Clinical Assessment

Psychological assessment was identical to that of study I, but a neurological disability assessment was added, using a questionnaire that addresses the different aspects considered in the Expanded Disability Status Scale (51).

The VAS was assessed directly before (VAS1) and after (VAS2) stimulation and after the vigilance task (VAS3).

#### Stimulation

Stimulation was applied in the same way as in study I. In the verum, and in the placebo group, the anode (5 cm × 7 cm) was placed over the right parietal cortex (P4), and the cathode (5 cm × 7 cm) was placed over the contralateral forehead.

#### Statistical Analysis

Analyses of variances were used to check for group differences in performance on the vigilance task (median RT, errors) and in subjective fatigue (VAS). The treatment condition (VERUM vs. PLACEBO) was defined as between-subject factor TREATMENT. To control for a possible effect of the level of cognitive fatigue on the efficacy of stimulation, we divided patients into those suffering from severe cognitive fatigue and those suffering from mild to moderate cognitive fatigue, based on the median cognitive fatigue score of the FSMC. Accordingly, we included the cognitive fatigue level (MILD/MODERATE vs. SEVERE) as a second between-subject factor FATIGUE. We compared RTs and number of errors from the first (BEGIN) and last (END) 5 min of the vigilance task and defined TIME (BEGIN vs. END) as within-group factor. Changes in subjective fatigue level were analyzed using the VAS1, VAS2, and VAS3 as within-group factor TIME. Significant main or interaction effects were tested further using *post hoc* tests.

### Results

#### Demographic and Psychological Data

**Figure 4:**
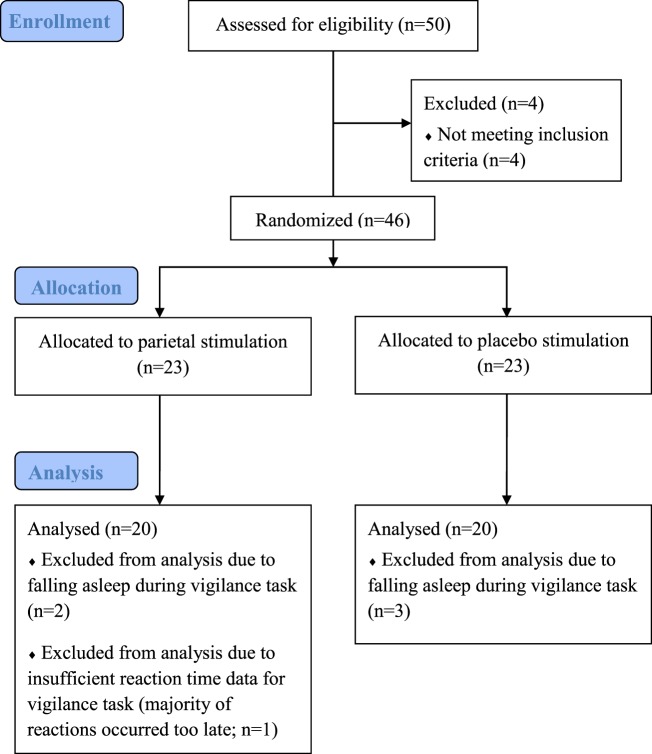
**Flowchart of study II**.

All patients had at least a cognitive fatigue scale value of 22 on the FSMC. Five patients were excluded from the analysis because they fell asleep during the vigilance test. Three of them belonged to the placebo group, two to the verum group. Another patient was excluded from the analysis because of missing and/or insufficient RT data for most 5-min intervals of the vigilance task (Figure 4). Mildly to moderately cognitively fatigued and severely cognitively fatigued patients of the placebo and the stimulation group did only differ in fatigue assessments, with mildly to moderately fatigued patients scoring significantly lower on these assessments than severely cognitively fatigued patients (Table 4).

**Table 4:** **Demographic, psychological, and reaction time data of multiple sclerosis patients (study II)**.

	Study II			
	Verum		Placebo	
	Mild/moderate CF	Severe CF	Mild/moderate CF	Severe CF
Number of individuals	10	10	10	10
Age (SD)	51.8 (9.9)	50.9 (8.8)	47.1 (10.3)	46.5 (9.1)
Gender (male:female)	4:6	3:7	4:6	4:6
Disease duration in month (SD)	142.8 (75.2)	135.2 (86.3)	180.1 (136.5)	126.8 (101.8)
Symptom duration in month (SD)	228.0 (135.7)	276.7 (111.3)	223.9 (130.2)	186.9 (109.1)
Disease type (RRMS:CPMS)	5:5	3:7	4:6	3:7
Disease-modifying therapy (no:yes)	3:7	2:8	4:6	4:6
EDSS (SD)	4.0 (1.5)	4.8 (1.2)	3.4 (2.1)	4.5 (1.0)
FSS (SD)*^,^^a^	3.92 (1.16)	5.61 (0.93)	4.06 (1.60)	5.27 (1.33)
FSMC total score (SD)*^,^^a^^,^^b^^,^^c^^,^^d^	65.1 (7.1)	84.6 (8.6)	56.3 (8.0)	79.3 (11.6)
FSMC cognitive score (SD)*^,^^a^^,^^b^^,^^c^^,^^d^	31.6 (3.5)	42.3 (5.1)	27.2 (3.8)	40.8 (5.0)
FSMC motor score (SD)*^,^^a^^,^^b^^,^^c^	33.5 (4.7)	42.3 (4.4)	29.1 (4.5)	38.5 (6.9)
ESS (SD)	7.8 (4.5)	11.2 (4.2)	9.8 (3.8)	9.0 (3.4)
PSQI (SD)	7.2 (4.2)	10.4 (4.5)	6.8 (3.3)	8.2 (3.3)
BDI (SD)	11.9 (9.8)	14.8 (8.4)	10.3 (7.2)	11.4 (6.6)
VAS1 (before tDCS) (SD)	43.8 (24.9)	52.8 (26.5)	30.8 (19.1)	39.5 (24.9)
VAS2 (after tDCS) (SD)	32.3 (20.6)	44.0 (27.1)	20.0 (12.3)	38.0 (26.7)
VAS2 (before vigilance task) (SD)	32.3 (20.6)	44.0 (27.1)	20.0 (12.3)	38.0 (26.7)
VAS3 (after vigilance task) (SD)*^,^^c^	43.9 (28.1)	63.2 (24.9)	28.7 (22.8)	53.9 (25.1)
RT (SD) vigilance task (first 5 min)	591.5 (±105.5)	549.2 (±117.3)	473.4 (±120.1)	519.5 (±129.8)
RT (SD) vigilance task (last 5 min)	548.6 (±92.5)	550.9 (±92.9)	553.2 (±106.8)	507.6 (±89.1)

*p < 0.05.

*^a^Mildly/moderately cognitively fatigued verum vs. severely cognitively fatigued verum*.

*^b^Mildly/moderately cognitively fatigued placebo vs. severely cognitively fatigued placebo*.

*^c^Mildly/moderately cognitively fatigued placebo vs. severely cognitively fatigued verum*.

*^d^Mildly/moderately cognitively fatigued verum vs. severely cognitively fatigued placebo*.

*BDI, Beck Depression Inventory; CF, cognitive fatigue; CPMS, chronic progressive multiple sclerosis; EDSS, Expanded Disability Status Scale; ESS, Epworth Sleepiness Scale; FSMC, Fatigue Scale for Motor and Cognition; FSS, Fatigue Severity Scale; PSQI, Pittsburgh Sleep Quality Index; RRMS, relapsing–remitting multiple sclerosis; RT, reaction time; VAS, Visual Analog Scale*.

#### Performance on the Vigilance Task

**Figure 5:**
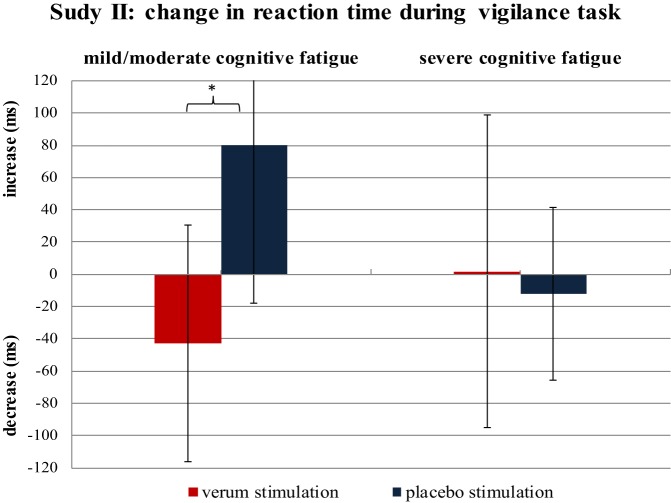
**Change in reaction time from the first to the last 5 min of the vigilance task**. The difference in reaction time is calculated separately for mildly cognitively fatigued and severely cognitively fatigued multiple sclerosis patients belonging to the verum and placebo stimulation group (each group *n* = 10). *p = 0.012.

The ANOVA on RTs revealed a significant interaction effect for TREATMENT × TIME (*p* = 0.043; *F* = 4.381) as well as for TREATMENT × FATIGUE × TIME (*p* = 0.013; *F* = 6.826). While the verum group showed a decrease in RT with time-on-task [begin: 570.3 ms (±110.7); end: 549.7 ms (±90.3)], the placebo group presented an increase [begin: 496.4 ms (±124.0); end: 530.4 ms (±98.5)]. When considering the different fatigue groups, the positive effect of stimulation on RTs was more pronounced in MS patients suffering from mild to moderate fatigue than in severely cognitively fatigued patients (Figure 5). While mildly to moderately cognitively fatigued MS patients of the stimulation group presented a moderate decrease in RTs with time-on-task [begin: 591.5 ms (±105.5); end: 548.6 ms (±92.5); *p* = 0.097], severely cognitively fatigued patients of the stimulation group showed hardly any change in RTs [begin: 549.2 ms (±117.3); end: 550.9 ms (±92.9); *p* = 0.957]. The change in RT differed significantly between mildly to moderately cognitively fatigued MS patients of the placebo and the stimulation group (*p* = 0.012). While those patients belonging to the stimulation group showed a moderate decrease in RTs with time-on-task (*p* = 0.097), those belonging to the placebo group showed a significant increase in RTs (*p* = 0.030). There was no correlation between the level of depression and the performance on the vigilance task.

The ANOVA on the number of errors did not reveal any significant main or interaction effects.

#### Subjective Fatigue

The ANOVA on the VAS data revealed significant differences between fatigue groups (*p* = 0.017; *F* = 6.236). *Post hoc* tests showed that patients suffering from severe cognitive fatigue scored significantly higher on the VAS data assessed before (*p* = 0.044) and after the vigilance task (*p* = 0.009) than patients with mild to moderate cognitive fatigue. Moreover, the ANOVA revealed a significant effect of TIME (*p* < 0.001; *F* = 9.006). All groups presented a decrease in their subjective fatigue level when comparing VAS data assessed before (VAS1) and after (VAS2) stimulation. The comparison between the VAS data assessed before (VAS2) and after (VAS3) vigilance performance demonstrated an increase in subjective fatigue level for all groups. There were no significant interaction effects between different groups (TREATMENT; FATIGUE) and the factor TIME.

### Discussion

Our results suggest that anodal stimulation over the right parietal cortex exerts a positive effect on the decrement of RT during a vigilance task. Patients who received anodal stimulation over the right parietal cortex showed a decrease in their RT with time-on-task, whereas patients of the placebo group presented an increase in their RT with ongoing time. The positive effect of stimulation was only observed in patients suffering from mild to moderate cognitive fatigue, not in severely cognitively fatigued patients. All groups presented an increase in subjective fatigue after the vigilance task. This increase was independent of stimulation, suggesting a dissociation between behavioral and subjective measures of fatigue. This assumption is compatible with our model for MS-related fatigue (52).

### General Discussion

The results of study I and study II indicate that anodal stimulation over the right parietal cortex counteracts the performance decrement during a vigilance task, which is primarily reflected in the RT data. In both studies, individuals receiving anodal stimulation over the right parietal cortex showed a decrease in RT with time-on-task, in comparison to individuals receiving frontal or placebo stimulation. In MS patients, the positive effect of parietal stimulation was restricted to patients suffering from mild to moderate cognitive fatigue.

The positive effect of right parietal stimulation is likely to be ascribed to enhanced excitability in the parietal cortex resulting from anodal tDCS. Neuroimaging studies demonstrated that vigilance relies on a right-hemispheric frontal, parietal, thalamic, and brainstem network (18–20), and previous studies frequently reported an association between vigilance decrement and a decrease of neural activity in the thalamus, the right-hemispheric ventro–dorsolateral frontal, parietal, and temporal cortex (11, 22–24). Moreover, several studies on structural correlates of MS-related fatigue demonstrated an association between fatigue and atrophy within the parietal lobe (27, 28, 31, 32, 34). Therefore, anodal excitability-enhancing tDCS over the right parietal cortex may counteract vigilance decrement by enhancing processing in the parietal cortex.

The observed decline in RT appeared at the end of the vigilance test, after stimulation was stopped. Previous studies demonstrated that the effect of tDCS persists minutes to hours after stimulation ended, depending on stimulation parameters and cortical areas that have been stimulated (53). Therefore, it seems that the decline in RT may be ascribed to after-effects of parietal stimulation.

Our results indicate that stimulation is more effective in MS patients suffering from mild to moderate cognitive fatigue than in those suffering from severe cognitive fatigue. The four composed groups of MS patients did not differ in the severity of depression. Hence, we assume that the obtained results are not due to differences in the level of depression. Severely fatigued patients might suffer from increased atrophy or a larger neurotransmitter imbalance in stimulated brain areas, which might explain the reduced effect of stimulation in this group. Several MRI studies on structural correlates of MS-related fatigue point to a positive correlation between fatigue level and atrophy within parietal brain regions (28, 32, 34). Probably, duration and intensity of stimulation were not sufficient to exert a positive effect in severely cognitively fatigued patients. However, these considerations need to be investigated further.

In contrast to our hypothesis, stimulation of the right frontal cortex did not reveal positive effects on vigilance performance. Healthy individuals receiving frontal stimulation performed even worse with respect to RT than the placebo group. This finding is in line with results obtained by Nelson and colleagues (54). These authors compared the effect of anodal stimulation over the left frontal cortex (anode F3; cathode F4) with that of anodal stimulation over the right frontal cortex (anode F4; cathode F3) on vigilance performance and found no effect on RT. Both groups presented an increase in RT with time-on-task. The negative effect of right frontal stimulation that we observed might point to a possible effect of stimulation on task-irrelevant information processing. Frontal stimulation might induce changes in neural excitability resulting in increased neural processing within brain areas that process interoceptive information. Consequently, frontal stimulation might cause interoceptive interference distracting cognitive processes causing an increase in RT. Moreover, stimulation of the frontal cortex might increase mind wandering. This assumption is supported by Axelrod and colleagues (55) who demonstrated that stimulation of the frontal cortex has the potential to increase the propensity to mind wander. Anodal excitability-enhancing stimulation over the right frontal cortex might also increase inter-hemispheric inhibition of the left frontal cortex resulting in slower motor responses and performance decline. However, these interpretations are rather speculative, and we cannot say which mechanisms cause the vigilance decrement in the frontal stimulation group, but right frontal anodal tDCS appears to exert negative effects on RTs during a vigilance task.

Healthy controls scored in the range of mild pathological fatigue concerning the FSMC. However, according to the results on the FSS, healthy controls did not display a pathological fatigue level. This apparent discrepancy between the two questionnaires might well be due to different definitions of the cutoff value for pathological fatigue. Presumably, for the FSMC, the cutoff value for fatigue is set quite low to get a highly sensitive measure for fatigue. However, at the same time, the specificity of the questionnaire decreases. Such a difference between questionnaires may also explain why several healthy individuals displayed a pathological fatigue level on the FSMC.

Concerning vigilance performance, healthy controls made more errors at the beginning than at the end of the vigilance task. This finding is mainly due to the amount of commissions and might result from an uncertainty about the occurrence of target stimuli at the beginning of the task. With ongoing time, individuals perhaps become more familiar with the task characteristics, and consequently the number of commissions may decrease. Overall, the decrease in the number of errors with time-on-task indicates that the positive effect of parietal stimulation on RTs cannot be interpreted as a speed–accuracy tradeoff phenomenon.

A large increase in subjective fatigue was observed in all groups after the vigilance task, with severely cognitively fatigued patients presenting generally higher scores than mildly to moderately cognitively fatigued patients. Moreover, we excluded 5 out of 46 MS patients and 1 out of 52 healthy controls from the analysis because they fell asleep several times during the vigilance task. This demonstrates that the vigilance task is highly sensitive to fatigue in MS patients and has a great potential to induce increased subjective fatigue. Hence, a 20-min lasting vigilance task might present a valid diagnostic tool for objectively measuring cognitive fatigue in MS patients.

Stimulation had no effect on subjective fatigue. At first sight, this finding seems to contradict the behavioral data revealing a positive effect of parietal stimulation on the decrement of RTs. However, this finding is compatible with our model for fatigue in MS, which predicts exactly such a dissociation between the feeling and the behavioral characteristics of fatigue (52). According to this model, subjective fatigue in MS depends on peripheral inflammation and inflammation-induced activity changes within homeostatic brain regions like the hypothalamus, the anterior cingulated, or the insular cortex. Right parietal stimulation does not influence peripheral inflammation and resulting inflammation-induced activity changes within homeostatic brain regions. The behavioral aspects of fatigue seem to depend on focal brain atrophy and/or neurotransmitter imbalance affecting the alerting/vigilance network, including areas in the parietal lobe. Recently, we published a study on cortical underpinnings for fatigue in MS and demonstrated that fatigued patients present reduced cortical thickness within the right inferior parietal lobe (31). Consequently, excitability-enhancing anodal stimulation over the right parietal cortex should be able to increase excitability within the vigilance network and reduce vigilance decrement in fatigued MS patients, but it should not have any effects on the feeling of fatigue.

One limitation of our study is that the stimulation conditions in study I and study II slightly differed. While in study I, stimulation occurred during the first half of the 40-min lasting vigilance task, in study II, stimulation was applied before the 20-min lasting vigilance performance. Due to safety issues, we did not exceed a stimulation period of 20 min. We decided to apply tDCS in study II before the vigilance task, because in study I, differences between groups mainly arose after stimulation stopped. Moreover, we shortened the duration of the vigilance task in study II to minimize stress for fatigued MS patients. Nevertheless, we did find a positive effect of stimulation on vigilance decrement in study II. Furthermore, we did not assess vigilance performance before the stimulation. It would have been interesting to compare the assessed RTs with those measured before any intervention occurred. Moreover, evidence suggests that bilateral anodal stimulation might be even more effective than unilateral stimulation in enhancing cognitive functions, such as vigor activity (56). Two recent studies used bilateral anodal stimulation and found a positive effect of tDCS on subjective fatigue in MS (41, 43), suggesting that bilateral stimulation might produce even better effects on objective and subjective fatigue than unilateral stimulation.

In conclusion, our results indicate that anodal tDCS over the right parietal, but not over the right frontal cortex, can counteract the increase in RT that is frequently observed during a vigilance task. The positive effect of stimulation on the decrease in RTs was only observed in MS patients suffering from mild to moderate cognitive fatigue, suggesting that anodal tDCS might be a useful method to counteract fatigue-associated performance decline in MS patients that suffer from mild to moderate cognitive fatigue.

## Author Contributions

KH: planning and execution of the study, assessment of participants and patients, data analysis, and writing the manuscript. MB: help on patient assessment. KM: help on participant assessment. PE: help on writing the manuscript. AA: did provide us with a DC stimulator and help concerning methods and tDCS. AK: did help planning and sponsoring the study. HH: did help planning and executing the study, help on statistical analysis, and manuscript writing.

## Conflict of Interest Statement

The authors declare that the research was conducted in the absence of any commercial or financial relationships that could be construed as a potential conflict of interest. The reviewer MT and handling Editor declared their shared affiliation, and the handling Editor states that the process nevertheless met the standards of a fair and objective review.
